# The interchange of immunophilins leads to parallel pathways and different intermediates in the assembly of Hsp90 glucocorticoid receptor complexes

**DOI:** 10.1038/celldisc.2016.2

**Published:** 2016-04-05

**Authors:** Ima-obong Ebong, Victoria Beilsten-Edmands, Nisha A Patel, Nina Morgner, Carol V Robinson

**Affiliations:** 1 Department of Chemistry, Physical and Theoretical Chemistry Laboratory, University of Oxford, Oxford, UK

**Keywords:** Mass spectrometry, Hsp90, Glucocorticoid receptor, Immunophilins

## Abstract

Hormone receptors require participation of the chaperones Hsp40/Hsp70 to form client-transfer complexes with Hsp90/Hop. Interaction with the co-chaperone p23 releases Hop and Hsp70, and the immunophilin FKBP52 mediates transfer of the Hsp90-receptor complex to the nucleus. Inhibition of glucocorticoid receptor (GR) transport by FKBP51, but not by FKBP52, has been observed at the cellular level, but the subunit composition of the intermediates involved has not been deduced. Here we use mass spectrometry to show that FKBP51/52 form analogous complexes with GR/Hsp90/Hop/Hsp70/ATP, but differences emerge upon addition of p23 to client-transfer complexes. When FKBP51 is present, a stable intermediate is formed (FKBP51)_1_(GR)_1_(Hsp90)_2_(p23)_2_ by expulsion of Hsp70 and Hop. By contrast, in the presence of FKBP52, ejection of p23 also takes place to form the nuclear transfer complex (FKBP52)_1_(GR)_1_(Hsp90)_2_. Our results are therefore consistent with pathways in which FKBP51/52 are interchangeable during the early assembly reactions. Following interaction with p23, however, the pathways diverge with FKBP51 sequestering GR in a stable intermediate complex with p23. By contrast, binding of FKBP52 occurs almost concomitantly with release of p23 to form a highly dynamic transfer complex, primed for interaction with the dynactin transport machinery.

## Introduction

The involvement of molecular chaperones in the formation of active steroid receptor complexes was recognised more than 25 years ago [1] (reviewed in Pratt [2] and Smith and Toft [3]). Hsp90, in interaction with a steroid receptor, was found in complexes with one of the immunophilin related co-chaperones FKBP51, FKBP52, CyP40 or PP5 [4]. Chaperone interactions with the receptor are now known to maintain binding competence for ligands and transcription factors (reviewed in Pratt and Dittmar [5] and Echeverria and Picard [6]). In the case of the glucocorticoid receptor (GR), its hormone-binding ability is lost in the absence of Hsp90, but can be restored upon addition of Hsp90 and co-chaperones in a rabbit reticulocyte lysate [7]. At the molecular level, *in vitro* experiments have now shown that Hsp90 is not able to bind directly to GR but requires interaction with a range of co-chaperones (Hop, Hsp70, Hsp40 and p23) [8]. These four co-chaperones are defined as the minimal cohort required for assembly of Hsp90-receptor complexes with hormone-binding ability [8, 9]. The immunophilins (FKBP51 and FKBP52) are not required for this process but are invariably found in *ex vivo* complexes, along with Hsp90 and steroid receptors [4].

Despite their close functional and structural relationships, with 70% sequence similarity, divergent activity has been ascribed to FKBP51 and FKBP52. The two immunophilins have been shown to bind differently to Hsp90 through regions adjacent to their C-terminal tetratricopeptide repeat (TPR) domains [10]. The co-chaperone Hop also competes with FKBP52 for the two common MEEVD pentapeptide Hsp90 binding sites to form asymmetric complexes with the composition (Hsp90)_2_(Hop)_1_(FKBP52)_1_ with a closely affinity for the two TPR-binding proteins [4, 11, 12]. The X-ray structures of FKBP51 (1KT0) [13] and FKBP52 (1Q1C/1P5Q) [14] reveal common FK1 and FK2 domains that encompasses the functional peptidylprolyl isomerase active sites that promote folding and binds to the immunosuppressant FK506. Differences in the proline-rich loops that overhang the peptidylprolyl isomerase pockets, are critical for receptor regulation and are thought to be responsible for the divergent activity of both immunophilins. Unique properties of FKBP52 complexes include elevated hormone binding for GR and recruitment of the transport protein dynein [15]. FKBP51 by contrast is known to inhibit hormone binding to GR and prevention of nuclear targeting. These observations have led to proposals that FKBP52 is interchanged for FKBP51 during the late stages of the chaperone assembly pathway to enable localisation of GR [16, 17].

Recently, we defined a client-transfer complex in which two copies of Hsp70 and Hsp90 assemble with Hop to form a substrate-binding cleft for GR [18]. In this assembly, chemical crosslinking revealed an antiparallel arrangement of the two Hsp70s and led to the proposal that the first Hsp90-bound Hsp70 provides a binding interface for the incoming Hsp70:GR heterodimer. This asymmetric complex was found to be remarkably stable with a single Hop subunit incorporated (Hsp90)_2_(Hsp70)_2_(Hop)_1_(GR)_1_. Such a subunit arrangement leaves a vacant C-terminal MEEVD-binding site on Hsp90_2_ that could accommodate an additional TPR-binding immunophilin without perturbing other subunit interactions. How the transfer complex is affected by immunophilin binding and exchange is unclear. Also of particular interest are potential differences in the ways in which the immunophilin-bound client-transfer complexes respond to incoming p23, the co-chaperone shown to catalyse release of Hsp70 and Hop, in the presence of ATP, and to transfer the client to Hsp90 [18].

Here we consider at which stages it is possible to exchange FKBP51 and FKBP52, and whether or not the same intermediates are formed along parallel reaction pathways. To do this we use mass spectrometry (MS) to probe interactions between eight components involved in the assembly of GR:Hsp90 complexes, namely ligand-free GR (ligand-binding domain), Hsp90, Hop, Hsp70, p23, Hsp40, FKBP52 and FKBP51. We allow complexes to assemble in solution and define their composition using MS under non-denaturing conditions designed to preserve non-covalent interactions [19]. Starting with the Hsp90_2_:Hop:FKBP51/52 system we find that asymmetric complexes containing either of the immunophilins and Hop can form. We consider the effects of the interchange of immunophilins on the client-transfer complex (Hsp90)_2_(Hop)_1_(Hsp70)_2_(GR)_1_ and demonstrate significant differences in stability. Following challenge with the co-chaperone p23 we find that disassembly is impaired in the presence of FKBP51 yet productive when FKBP52 is bound, forming the key (Hsp90)_2_(FKBP52)_1_(GR)_1_ nuclear transfer complex. This latter complex is primed for further interaction with the dynactin machinery and subsequent transfer to the nucleus.

## Results

### FKBP52 and FKBP51 compete with Hop for binding sites in Hsp90

**Figure 1:**
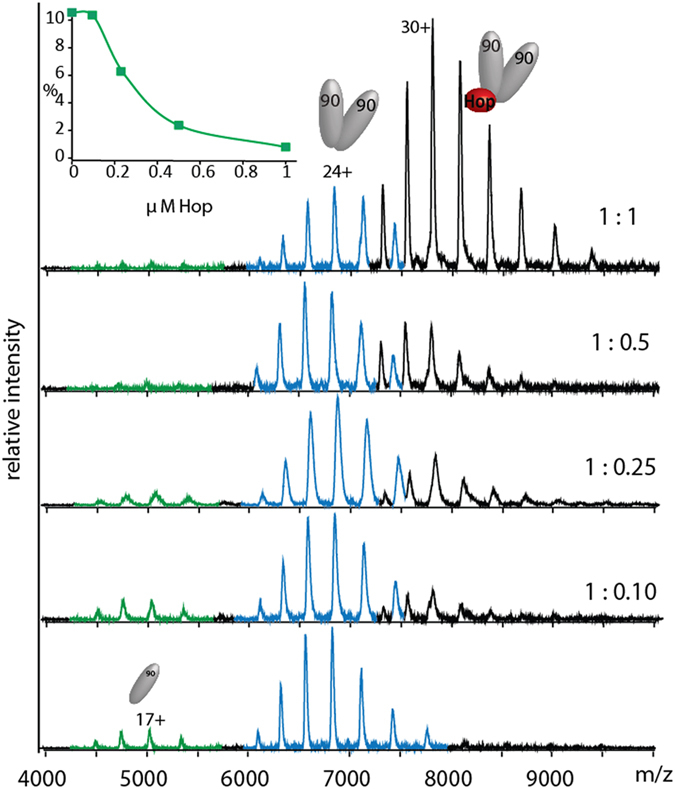
Binding of Hop to Hsp90. Increasing molar ratios of (Hsp90)_2_:Hop (1 μm:100 nm to 1 μm) from bottom to top lead to formation of the (Hsp90)_2_Hop complex. The lower spectrum is recorded in the absence of Hop. As the concentration of the free Hsp90 dimer is depleted (blue) by addition of Hop, the population of monomeric Hsp90 (green), is reduced. Inset: plot of the percentage signal intensity for monomeric Hsp90 against the concentration of Hop in solution. The percentage abundance was calculated from *Massign* [22].

Given that the two immunophilins FKBP51 and FKBP52 are thought to target the same MEEVD-binding sites of Hsp90 [20] and to compete with established binding of Hop (or Sti1 the yeast homologue) [21], we first considered binding of Hop to Hsp90. To a solution of Hsp90_2_ (1 μm) increasing concentrations of Hop (from 100 nm to 1 μm) were added and mass spectra recorded (Figure 1). All measured and calculated masses are noted (Supplementary Table S1). Peaks assigned to monomeric Hsp90 reduced in intensity as Hop is added in increasing quantities until at stoichiometric concentrations the predominant complex is (Hsp90)_2_(Hop)_1_. A plot of increasing concentrations of Hop against the fraction of Hsp90 in monomeric form shows that the population of (Hsp90)_1_ diminishes in line with the reduced concentration of free Hsp90 in solution. We conclude therefore that the presence of Hop in solution perturbs the monomer–dimer equilibrium by stabilizing the Hsp90 dimer in the (Hsp90)_2_(Hop)_1_ complex.

We next considered binding of FKBP51 and FKBP52 to Hsp90 at a range of concentrations from 1 to 3 μm. At a concentration of 1 μm Hsp90_2_ to 2 μm of both FKBP51 and FKBP52 we observed the anticipated statistical distribution of Hsp90 complexes with two immunophilins bound. Under the highest concentrations studied, Hsp90_2_ (3 μm) incubated with both FKBP51 and FKBP52 with a twofold molar excess (6 μm; Supplementary Figure S1), showed the same predominant complexes with approximately equal affinity of the two immunophilins. At this concentration the mass spectrum also shows that up to two molecules of each immunophilin FKBP51/52 bind to monomeric Hsp90. Given that a single MEEVD site is present in monomeric Hsp90, a single copy of FKBP51/52 was expected to bind. Interestingly, we observed the complexes (Hsp90)_1_(FKBP51)_2_, (Hsp90)_1_(FKBP51)_1_(FKBP52)_1_ and (Hsp90)_1_(FKBP52)_2_ implying a second binding site on monomeric Hsp90.

**Figure 2:**
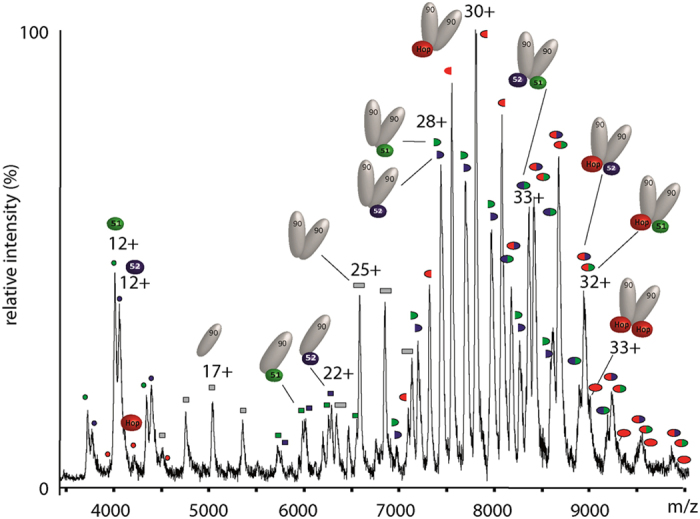
Mass spectrum of a solution of Hsp90_2_ incubated with Hop, FKBP51 and FKBP52 at equimolar concentrations (3 μm). Up to two TPR-binding proteins are able to bind to Hsp90_2_. The predominant complexes contain a single copy of Hop. A single copy of either of the immunophilins is also observed with Hop but complexes with two immunophilins or two Hop subunits also form with low intensity. A full assignment of this spectrum is available in Supplementary Figure S2.

Competition experiments were then carried out for (Hsp90)_2_-binding sites with the three TPR-containing proteins (FKBP52, FKBP51 and Hop) all at equimolar concentration (3 μm; Figure 2). The mass spectrum is complex but the dominant species are assigned to the three possible asymmetric ternary complexes (Hsp90)_2_(FKBP51)_1_(FKBP52)_1_, (Hsp90)_2_(FKBP51)_1_(Hop)_1_ or (Hsp90)_2_(FKBP52)_1_(Hop)_1_ using the programme Massign [22] (Supplementary Figure S2). These experiments show that any two of the three proteins (FKBP52, FKBP51 or Hop) can bind to the MEEVD-binding motif. Interestingly, the (Hsp90)_2_(Hop)_1_ complex predominates and very little free Hop is observed in solution, consistent with the higher affinity for Hop binding to (Hsp90)_2_ than the two immunophilins [12]. We also observed that the probability of forming a heterocomplex with two immunophilins is reduced given the higher affinity for Hop binding. Overall, this experiment confirms that binding of TPR proteins to the MEEVD residues in Hsp90_2_ is independent, but the higher affinity for Hop over the two immunophilins ensures the formation of complexes containing at least one Hop, which are therefore primed for interaction with Hsp70.

### Client transfer complexes with or without FKBP52 or FKBP51 react differently to p23

**Figure 3:**
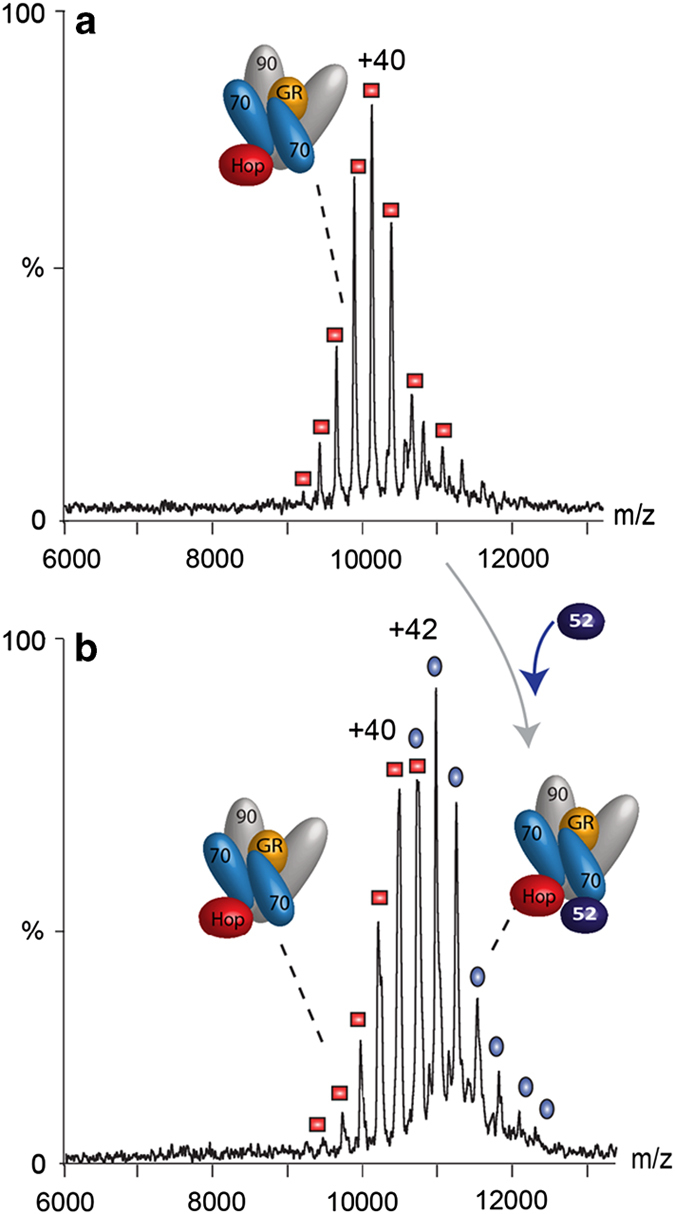
Formation of the hexameric client complex. (**a**) Incubation of stoichiometric ratios (all at 0.5 μm) of (Hsp70)_2_, GR, Hop and (Hsp90)_2_ in the presence of Hsp40 leads to the formation of a hexameric client-transfer complex as reported previously [18]. (**b**) On addition of an equimolar ratio of FKBP52 to this complex binding of a single FKBP52 subunit is observed (charge states labelled blue).

Having formed Hsp90 complexes in the presence of Hop and the immunophilins, the next stage is to see how FKBP51/52 influence complexes containing client proteins. When stoichiometric ratios of Hsp90_2_:Hsp70_2_:GR:Hop are incubated, in the presence of excess ATP, a hexameric client-transfer complex predominates as shown previously [18] (Figure 3). When p23 is added to the client-transfer complex (Hsp90)_2_(Hop)_1_(Hsp70)_2_(GR)_1_ the complex disassembles expelling both Hop and Hsp70_2_ to form (Hsp90)_2_(p23)_2_(GR)_1_ [18]. This complex containing both Hsp90 and GR, in the absence of Hsp70 and Hop, demonstrates that p23 has induced transfer of GR interactions from Hsp70 to Hsp90 and promoted disassembly of Hsp70 and Hop. The GR client protein has thus relinquished all interactions with Hsp70, in favour of Hsp90_2_, transferring the client from Hsp70 to Hsp90_2_.

Before adding the immunophilins to this reaction, we carried out control experiments in the presence of ATP and catalytic amounts of Hsp40_2_ to show that in the absence of the client protein (GR) p23 does not bind to Hsp90/Hsp70/Hop complexes (Supplementary Figure S3). We also probed the stability of the intermediate complex (Hsp90)_2_(p23)_2_(GR)_1_, and found it to be stable and long-lived in solution. To investigate the strength of its subunit interactions, we assessed the stability of (Hsp90)_2_(p23)_2_(GR)_1_ using collision-induced dissociation in the gas phase. MS/MS of an isolated charge state (32+) revealed loss of GR and formation of a stripped complex of (Hsp90)_2_(p23)_2_ (Supplementary Figure S4). Expansion of the peaks assigned to this stripped complex reveals peak-splitting corresponding to nucleotide binding, consistent with ATP turnover during this Hsp90:GR:p23 interaction. This GR binding was observed without a ligand and in all cases GR binding was observed following prior incubation with Hsp70/40. Given that p23 is smaller than GR, p23 might be expected to dissociate in preference to GR [23]. The ready expulsion of GR however suggests that p23 interaction interfaces are larger than those of the client protein.

Having established the stability and requirements for GR binding we explored the asymmetry of the client-transfer complex with the vacant MEEVD-binding site, which exists on the Hsp90 dimer. This vacant site could accommodate an additional TPR-binding co-chaperone such as an immunophilin. To test this hypothesis we incubated the same protein components in stoichiometric ratios Hsp90_2_:Hsp70_2_:GR:Hop in the presence of Hsp40_2_, but this time with FKBP52 (Figure 3). This led to the formation of a second complex, analogous to the client-transfer complex, but with an additional FKBP52 subunit-incorporated (Hsp90)_2_(Hop)_1_(Hsp70)_2_(GR)_1_(FKBP52)_1_.

**Figure 4:**
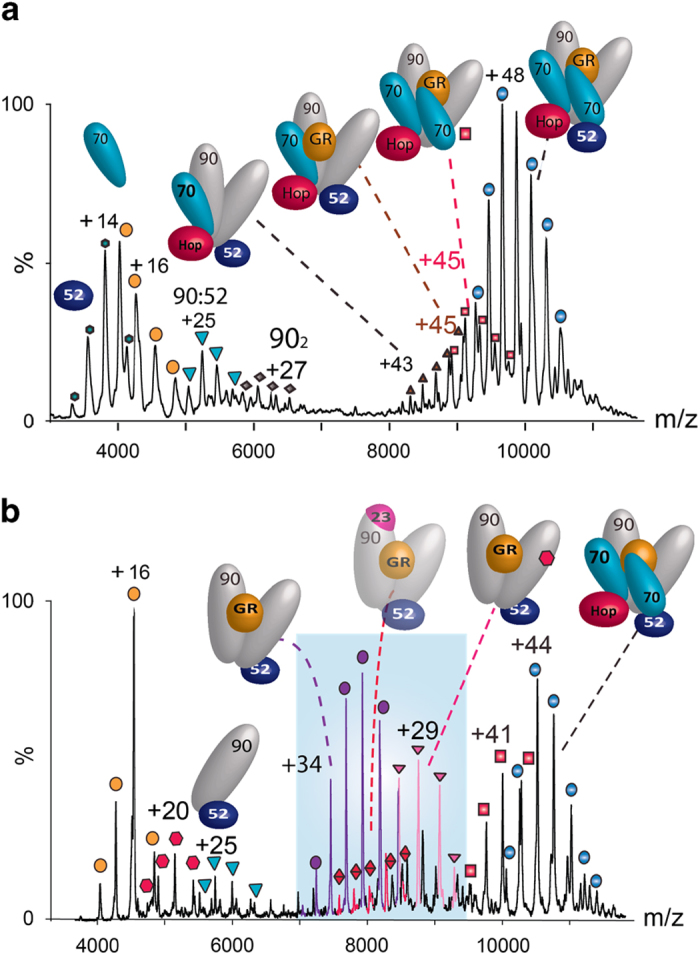
FKBP52 affects the formation of complexes formed after addition of p23. (**a**) Incubation of an excess of FKBP52 in a solution containing Hsp90_2_, Hsp70_2_, Hop, GR (all at 1 μm), Hsp40 (0.3) μM and ATP (200 μM) leads to the formation of the predominant complex (Hsp90)_2_(Hop)_1_(Hsp70)_2_(GR)_1_(FKBP52)_1_. (**b**) Addition of p23 to this complex leads to formation of (Hsp90)_2_(GR)_1_(FKBP52)_1_ in an extended charge state series (highlighted in blue) indicative of two conformations, the higher *m*/*z* series is consistent with nucleotide binding. A very low intensity series of peaks is assigned to a population of (Hsp90)_2_(GR)_1_(FKBP52)_1_(p23)_1_ (red diamonds). A full assignment of this spectrum is available in Supplementary Figures S6 and S7.

To explore the effect of FKBP52 on client-transfer complex formation, we incubated all the components simultaneously (Hsp70/Hsp90/Hop/GR/Hsp40/FKBP52/ATP) as opposed to addition of FKBP52 to the client-transfer complex once formed as carried out above. We again found that the client-transfer complex (Hsp90)_2_(Hop)_1_(Hsp70)_2_(GR)_1_(FKBP52)_1_ formed as the dominant species (Figure 4a). A lower population of the client-transfer complex without FKBP52 was also observed together with client-containing complexes with monomeric Hsp70 at very low intensity. To this mixture of complexes we then added stoichiometric quantities of p23. This led to the formation of a high population of (Hsp90)_2_(GR)_1_(FKBP52)_1_ (Figure 4b, highlighted in blue). Interestingly no complexes incorporating two copies of p23 were observed. This was a surprising result given that the incorporation of two p23 subunits in the absence of FKBP52 led to the formation of the predominant complex (Hsp90)_2_(GR)_1_(p23)_2_ [18]. The only p23-containing complex observed at low intensity was (Hsp90)_2_(p23)_1_(GR)_1_(FKBP52)_1_. The fact that this complex is only observed at such low intensity implies an intermediate state that is transiently populated in the presence of FKBP52, leading to a (Hsp90)_2_(GR)_1_(FKBP52)_1_ complex. If p23 is added to the client-transfer complex (Hsp90)_2_(Hop)_1_(Hsp70)_2_(GR)_1_ before FKBP52 the client-transfer complex disassembles to form the (Hsp90)_2_(GR)_1_(FKBP52)_1_ complex (Supplementary Figure S5). We conclude therefore that FKBP52 promotes dissociation of p23 from the (Hsp90)_2_(p23)_2_(GR)_1_ complex whatever the order of addition of the components, and ultimately gives rise to (Hsp90)_2_(GR)_1_(FKBP52)_1_, the complex primed for nuclear transfer.

**Figure 5:**
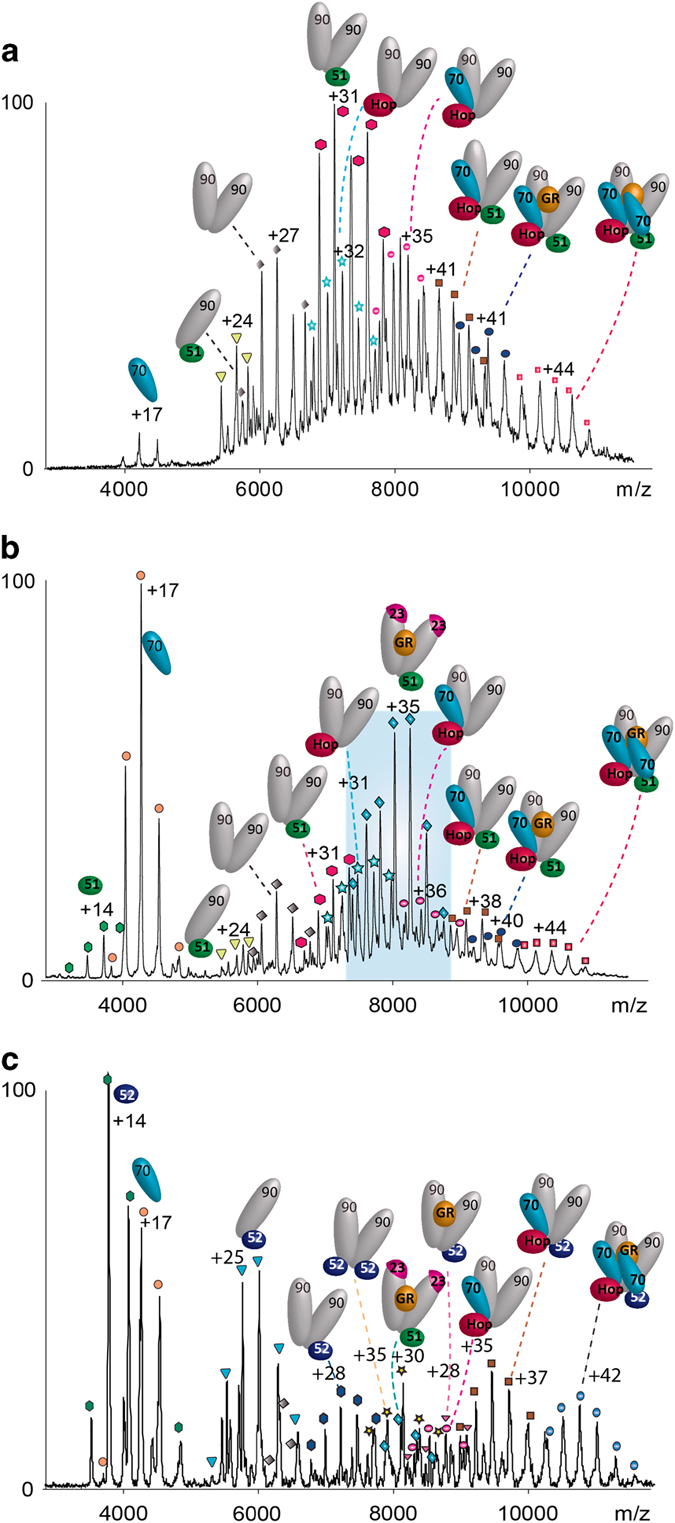
Formation of a client-transfer complex containing FKBP51 and subsequent challenge with p23 or FKBP52. (**a**) Incubation of an excess of FKBP51 in a solution containing Hsp90_2_, Hsp70_2_, Hop, GR (all at 1 μm), Hsp40 and ATP leads to the formation of the client-transfer complex (Hsp90)_2_(Hsp70)_2_(FKBP51)_1_(GR)_1_. (**b**) Following challenge of the client-transfer complex with p23 the predominant complex is (Hsp90)_2_(GR)_1_(FKBP51)_1_(p23)_2_. (**c**) Subsequent exchange of FKBP51 for FKBP52 leads to the formation of a series of complexes including (Hsp90)_2_(GR)_1_(FKBP52)_1_ the nuclear transfer complex and the client-transfer complex with exchange of FKBP51 for FKBP52. Interestingly no (Hsp90)_2_(GR)_1_(FKBP51)_1_ is formed in the presence of p23. A full assignment of this spectrum is available in Supplementary Figure S8.

We then compared the effects of incorporating FKBP51 rather than FKBP52 into the client-transfer complex before adding the p23 co-chaperone. In the presence of FKBP51 the corresponding predominant complex was (Hsp90)_2_(FKBP51)_1_ implying that complexes containing GR are destabilised by the presence of FKBP51 (Figure 5a). A small population of (Hsp90)_2_(GR)_1_(FKBP51)_1_ was formed however (Figure 5a), although without interaction with p23, this complex would not be capable of hormone binding [8]. The analogous client-transfer complex was obtained (Hsp90)_2_(Hop)_1_(Hsp70)_2_(GR)_1_(FKBP51)_1_ albeit at low population. Our results allow us to conclude that the client-transfer complex containing one vacant MEEVD-binding site can be occupied by either FKBP51/52 but the GR-containing complexes are more stable in the absence of an immunophilin or in the presence of FKBP52 rather than FKBP51.

We then added p23 to the FKBP51 solution containing the client-transfer complex (Hsp90)_2_(Hop)_1_(Hsp70)_2_(GR)_1_(FKBP51)_1_. The complex (Hsp90)_2_(GR)_1_(FKBP51)_1_, present in the absence of p23, dissociates indicating that this complex would not act as the corresponding nuclear transfer complex *in vivo* when p23 is present. Rather, this complex is a stable off-pathway intermediate present only in the absence of p23. We found that rather than the equivalent nuclear transfer complex observed with FKBP52 (Hsp90)_2_(GR)_1_(FKBP52)_1_, we formed a stable complex of the subunit composition (Hsp90)_2_(GR)_1_(p23)_2_(FKBP51)_1_ with two p23 subunits remaining incorporated (Figure 5b). Some disassembly of the client-transfer complex was also evident with a relatively high population of Hsp70 monomer and formation of (Hsp90)_2_ complexes with Hop and FKBP52. A population of a stable client-transfer complex with FKBP51 remains; but no productive nuclear transfer complex, analogous to (Hsp90)_2_(GR)_1_(FKBP52)_1_ which is formed with FKBP52, was observed with FKBP51.

As (Hsp90)_2_(GR)_1_(p23)_2_(FKBP51)_1_ is stable in solution while the analogous complex with FKBP52 is not stable, it is of interest to monitor the possible exchange of FKBP51 for FKBP52 in this complex. To investigate this we incubated the components Hsp90/Hsp70/Hop/GR/Hsp40/p23/FKBP51 as previously mentioned (Figure 5b). We then challenged the resulting mixture with excess FKBP52 (Figure 5c). On addition of FKBP52, FKBP51 is largely displaced from all complexes, a diminished population of the previously dominant complex (Hsp90)_2_(p23)_1_(GR)_1_(FKBP51)_1_ remains. That this complex reduces in intensity considerably implies that the addition of FKBP52 results in displacement of both FKBP51 and p23. Interestingly a predominant complex formed in this reaction is monomeric (Hsp90)_1_(FKBP52)_1_ suggesting that excess FKBP52 disrupts the complex and forms a stable dissociation product with monomeric Hsp90. This demonstrates that the previously dominant nuclear transfer complex (Hsp90)_2_(FKBP52)_1_(GR)_1_ can form in this reaction but at reduced intensity consistent with a less productive route compared with FKBP52 being present before challenge with p23.

Interestingly, the dominant nuclear transfer complex (Hsp90)_2_(FKBP52)_1_(GR)_1_ exhibits two charge state distributions (Figure 4b and Supplementary Figure S4). Charge state distributions are sensitive to conformations of proteins in solution. When multiple charge states coexist in the same spectrum they imply that more than one conformation of a protein or subunit is present [24]. Close inspection of the extended charge state series reveals that additional nucleotide is bound to the lower charged species at higher mass to charge (*m*/*z*) values. We assign this extended charge state distribution to open and closed conformations of Hsp90 within the (Hsp90)_2_(FKBP52)_1_(GR)_1_ complex, the closed conformation stabilized by nucleotide binding to (Hsp90)_2_ observed at higher *m*/*z* values. The fact that these two conformations coexist, but are not observed when p23 is bound (Figure 5b, blue), is consistent with p23 locking Hsp90 into the closed conformation, as shown crystallographically for (Hsp90)_2_(p23)_2_ [25]. The increased dynamics observed here in the presence of FKBP52 arises from the freedom of movement of Hsp90 in the absence of p23 and is in contrast to the stability of the closed ATP-bound form in the presence of p23 and FKBP51 (Figure 5b, blue).

## Discussion

In the absence of client proteins the Hsp90 chaperone assembly operates as a flexible assembly platform [26] that is regulated by co-chaperones [21] and able to bind to FKBP51 and FKBP52. Similar affinities were deduced for the two immunophilins, which compete with Hop for MEEVD-binding sites on Hsp90, consistent with earlier findings wherein Hsp90 was shown to bind concomitantly to the immunophilins and Hop [11, 27]. The affinity of Hop is higher than that of the immunophilins, as shown here and reported previously [12], leading to a greater population of heterocomplexes containing (Hsp90)_2_(Hop)_1_(Hsp70)_1_(FKBP). At high immunophilin concentrations monomeric Hsp90 complexes of the form (Hsp90)_1_(FKBP51)_2_, (Hsp90)_1_(FKBP51)_1_(FKBP52)_1_ and (Hsp90)_1_(FKBP52)_2_ were observed. These observations were surprising as monomeric Hsp90 contains only one MEEVD site and hence only a single immunophilin would be expected to bind. A second immunophilin-binding site on monomeric Hsp90 is implicated by these interactions. An additional N-terminal TPR-binding site in Hsp90, accessed by a subset of co-chaperones including FKBP52, has been suggested previously to enable coordinated binding of co-chaperones to N- and C-terminal sites of Hsp90 [28]. It could be therefore that FKBP51 and FKBP52 are binding to the C-terminal site and this postulated N-terminal binding site.

Our results for the binding of immunophilins to client-transfer complexes have shown that binding of FKBP51 and FKBP52 to MEEVD sites is similar with respect to their interactions with Hsp90 in the absence of client, but that this correspondence does not hold in the presence of GR or p23. Stable client-transfer complexes form with FKBP52, or in the absence of immunophilins, while incorporation of FKBP51 perturbs interactions and reduces stability of the client-transfer complex triggering the dissociation of the two Hsp70s and Hop. Two p23 subunits are able to bind stably in the presence of FKBP51, yet when FKBP52 is present the dominant complex does not contain p23 and a complex incorporating one p23 only at low intensity is observed. In the case when FKBP51 is present initially, addition of FKBP52 promotes disassembly of p23 and FKBP51. This series of events leads to the formation of the (Hsp90)_2_(GR)_1_(FKBP52)_1_ transfer complex, albeit at lower intensity than the direct route, without immunophilin exchange, and with considerable dissociation of the client-transfer complex.

**Figure 6:**
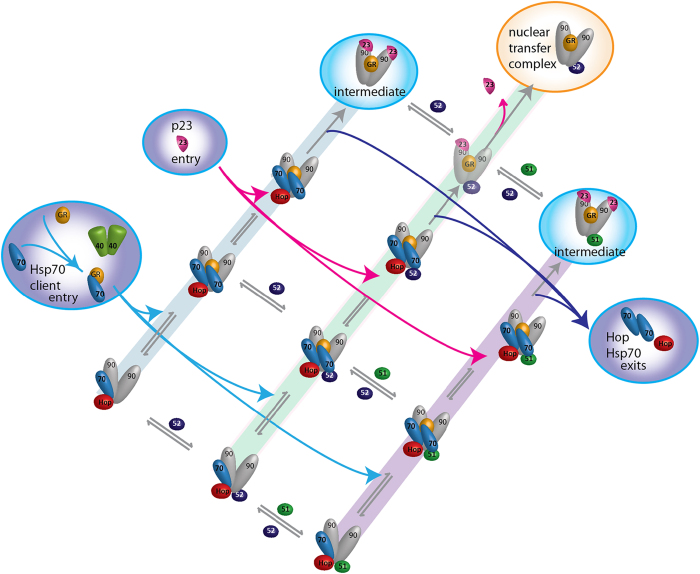
Schematic representation of the interconversion of complexes observed and the intermediates characterised. Starting with the (Hsp90)_2_(Hop)_1_(Hsp70)_1_ complex, introduction of the immunophilins (FKBP51/52) leads to parallel reaction pathways in which the immunophilins are interchangeable and not required for the formation of these early complexes. Similarly three corresponding client-transfer complexes are formed when a second copy of Hsp70 and the GR client enter the reaction cycle. Interesting differences are observed when p23 is added to the client-transfer complex in the absence of the immunophilin or with FKBP51, two copies of p23 are incorporated with concomitant loss of Hsp70 and Hop. By contrast no stable complex with two p23 subunits is observed in the presence of FKBP52; expulsion of Hsp70, Hop and p23 occur with a low population of a complex incorporating only one p23 subunit. This central pathway leads to formation of the productive nuclear transfer complex, which is highly dynamic and primed for interaction with dynactin.

Although the precise mechanism by which FKBP51/ FKBP52 exchange and influence p23-binding and formation of the nuclear transfer complex in the cellular environment is not clear, it is evident that for large parts of the assembly reaction, parallel pathways with different immunophilins and interchange between them is possible (Figure 6). It is also important to consider how cellular concentrations of many of the chaperones and co-chaperones might affect immunophilin interchange. Although cellular concentrations are difficult to define the μm concentrations used here are in line with cellular concentrations defined previously (~10 μm for Hsp70 monomer, ~3 μm for Hop monomer and ~5 μm for Hsp90 dimer) [29]. However these concentrations were found to be altered following perturbation of the cell [29, 30]. The parallel pathways defined here would be dependent on local immunophilin concentrations and could be important for efficiency as complex assembly and disassembly could then occur with a minimum number of steps. The key difference at the end of the parallel pathways occurs when the single copy of p23, bound to the FKBP52-containing complex, dissociates leaving the nuclear transfer complex (Hsp90)_2_(GR)_1_(FKBP52)_1_ as the dominant product. This complex exhibits high conformational dynamics. In contrast if no FKBP52 is bound, two p23 subunits are stably incorporated and only dissociate upon binding of FKBP52.

Our findings are supported by *in vivo* observations that p23, in association with Hsp90, is co-precipitated from cells more readily with FKBP51 than with FKBP52 [31]. This is also in line with our observation that FKBP51 coexists in stable association with p23-containing Hsp90 heterocomplexes. Structural differences between FKBP51 and FKBP52 may allude to their differing functions. Full-length crystal structures have been solved for FKBP51 and for overlapping segments of FKBP52. The structures revealed are largely similar; however, FKBP51 adopts a more rigid compact conformation than FKBP52, which has an extended domain alignment [14]. In addition FKBP52 contains a flexible hinge region between its FK506-binding domain and FKBP-like domain [32]. Together the differences in these domain orientations and higher flexibility in FKBP52 could account for the differences seen in binding of p23 when FKBP51 or FKBP52 are present.

We have shown here that exchange of immunophilins can be regulated, in the presence of GR, based on the greater stability of FKBP52 client-transfer complexes. The formation of analogous complexes, both in the absence of immunophilins and in the presence of FKBP51, but not FKBP52, presents an important distinction between the immunophilins. A number of explanations may rationalise this differing behaviour at the molecular level. The observation that both immunophilins (FKBP51/52) are able to bind to monomeric Hsp90 implies a second binding site on Hsp90, possibly located at the N-terminus of Hsp90 [28]. This binding of the immunophilins to an N-terminal binding site on Hsp90 would position FKBP51/52 in close proximity to the p23-binding site such that modulation of p23–Hsp90 interactions would be possible. It is interesting to speculate that FKBP52 destabilises the p23–Hsp90 interaction from this position, in a way that FKBP51 is unable to access, in this way ensuring efficient formation of the essential nuclear transfer complex, which is the overall goal of the chaperone-client pathway.

## Materials and methods

### Protein preparation

All proteins used were human with the exception of yeast Hsp40 (ydj1). Proteins were provided by Sophie Jackson and David Agard’s labs and were expressed and purified as previously described [33]. In this study glucocorticoid ligand-binding domain construct was used and consisted of residues 521–777 with phenylalanine F602S mutation to enhance solubility. The yeast Hsp40 (ydj1) and the GR ligand-binding domain are referred to as Hsp40 and GR, respectively, for simplicity.

### *In vitro* assembly of Hsp90–client complexes

Protein complexes were assembled by incubating the proteins in binding buffer 30 mm HEPES, 50 mM KCl, 2 mm dithiothreitol, pH 7.5.

Unless stated otherwise, all proteins were added at an equimolar concentration of 1 μm, except Hsp40 (0.3 μm). Where nucleotide was required for binding, the nucleotide was added to a final concentration of 200 μm, and for p23 experiments 300 μm nucleotide was added.

To assemble the late complexes, FKBP and p23-containing complexes were formed by pre-forming Hsp90 heterocomplexes as stated above to assemble the Hsp90–client complexes. For FKBP52 the point of addition was varied. It was either added to the system from the start or 30 min afterwards (after 30 min delay time from the assembly start). For p23 incorporation in the late complex, the Hsp90–client complexes were first assembled and 300 μm ATP magnesium acetate was then added to the assembly after prior incubation for 30 min together with 1 μm p23 and incubated for a further 15 min. After formation, all complexes were buffer exchanged into 100 mM ammonium acetate pH 7.5 before MS analysis using micro Bio-Spin columns (Bio-Rad Laboratories, Hemel Hempstead, UK) or Vivaspin 10 kDa MWCO (Sartorius, Goettingen, Germany).

### MS analysis of complexes

All spectra shown here were obtained on a QToF II mass spectrometer (Waters, Manchester, UK) suitable for analysis of high-mass complexes [34]. For MS analysis, 2.5 μl of the solution containing the complex was introduced using a gold-plated capillary needle (Harvard Apparatus, Holliston, MA, USA) into the instrument. Spectra were acquired in the positive-ion mode, and all instrument conditions were kept constant where concentration effects were being monitored. Typical instrument parameters were as follows: capillary voltage 1.7–1.8 kV, cone voltage 100 V, extractor 5 V, collision voltage 100 V, backing pressure 9.65×10^−3^ mbar, analyser pressure 4×10^−4^ mbar and ToF 2.71×10^−6^ mbar. For MSMS experiments, collision voltages varied up to 200 V. All data shown were acquired and processed with MassLynx V4.1 (Waters, Manchester, UK), with minimal smoothing and no background subtraction. All recorded mass spectra were calibrated externally using caesium iodide prepared in water.
