# Supplementary material for: The interchange of immunophilins leads to parallel pathways and different intermediates in the assembly of Hsp90 glucocorticoid receptor complexes
Source: Cell Discov. 2016 Apr 5;2:16002–. doi: 10.1038/celldisc.2016.2 (PMC4849472; doi:10.1038/celldisc.2016.2)
Supplement: Supplementary information [file celldisc20162-s1.pdf]

Supplementary information

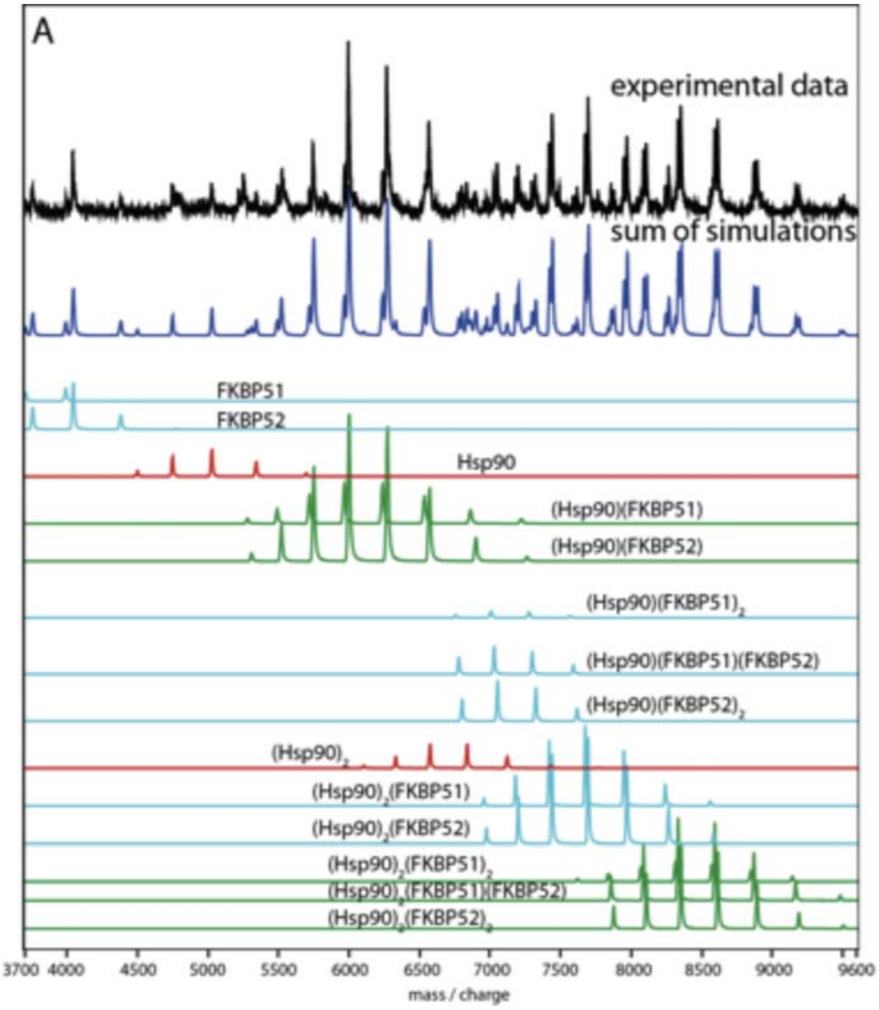

Figure S1 Mass spectrum of a 3 µM solution of $Hsp90_2$ incubated with FKBP51 and FKBP52 at 6 µM concentrations. This spectrum illustrates the mass spectral assignment protocol (Massign) applied throughout [1]. In this process the individual mass spectrum assigned to a protein complex is simulated from its mass to charge ratios, observed experimentally, as a Gaussian charge state distribution. The intensities of the individual component spectra are then adjusted and summed to mirror the experimental dataset. This process is iterated until the optimal fit between the experimental data and the simulated spectrum is achieved. Interestingly contributions from monomeric and dimeric Hsp90 bound to one or two immunophilins are revealed in this process.

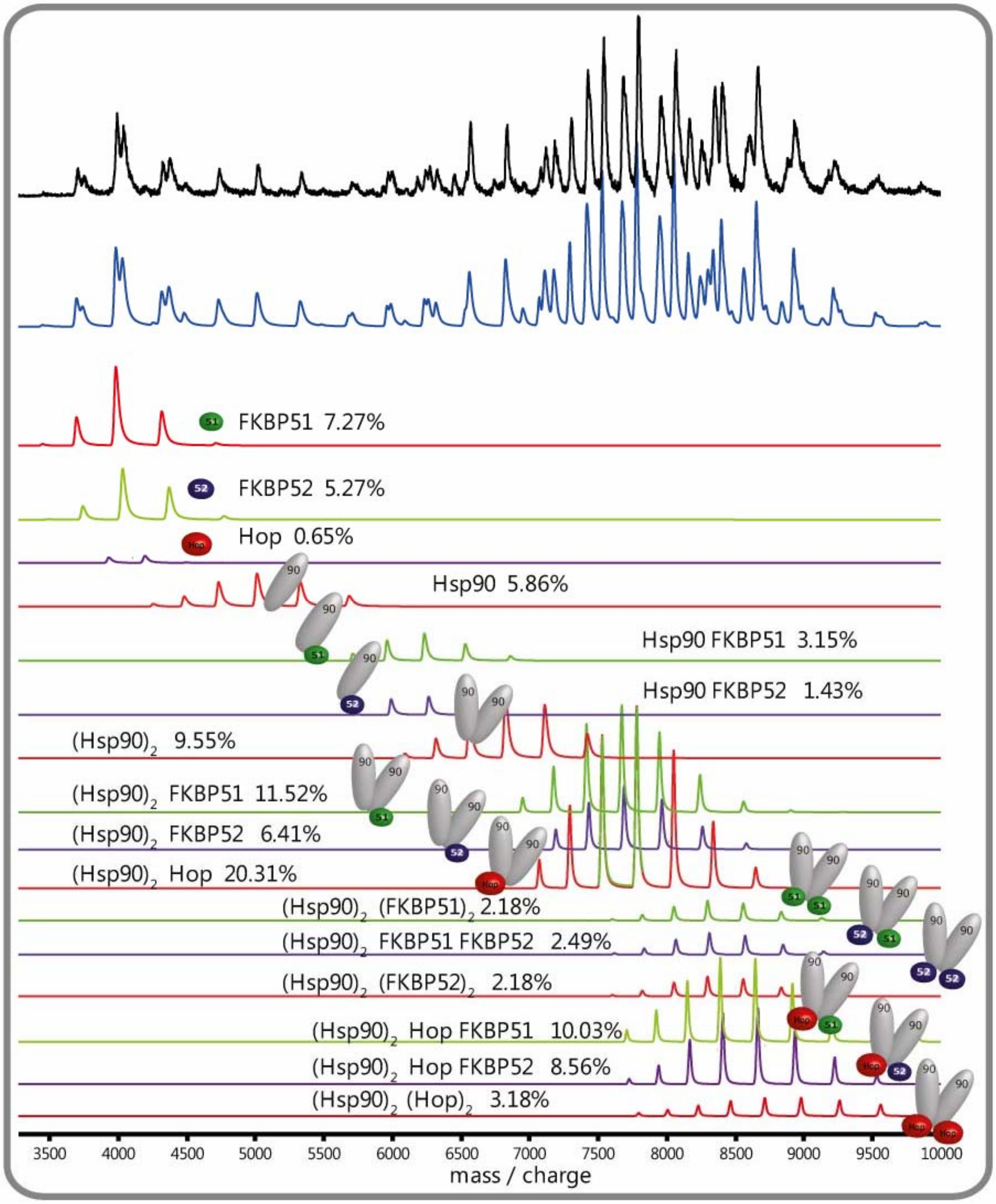

Figure S2 Assignment of the Hsp90 FKBP51/52/Hop spectrum and relative intensities of the complexes formed deduced by the program *Mass*ign [1]. Approximately equal affinities can be deduced for the two FKBP proteins however a higher affinity is apparent for Hop binding to both monomeric and dimeric forms of Hsp90 in accord with our earlier experiments. [2] We explored these interactions at two different protein concentrations but with the same ratio of component proteins, as reported in Table S2. We found no change in the complexes formed at the two different concentrations.

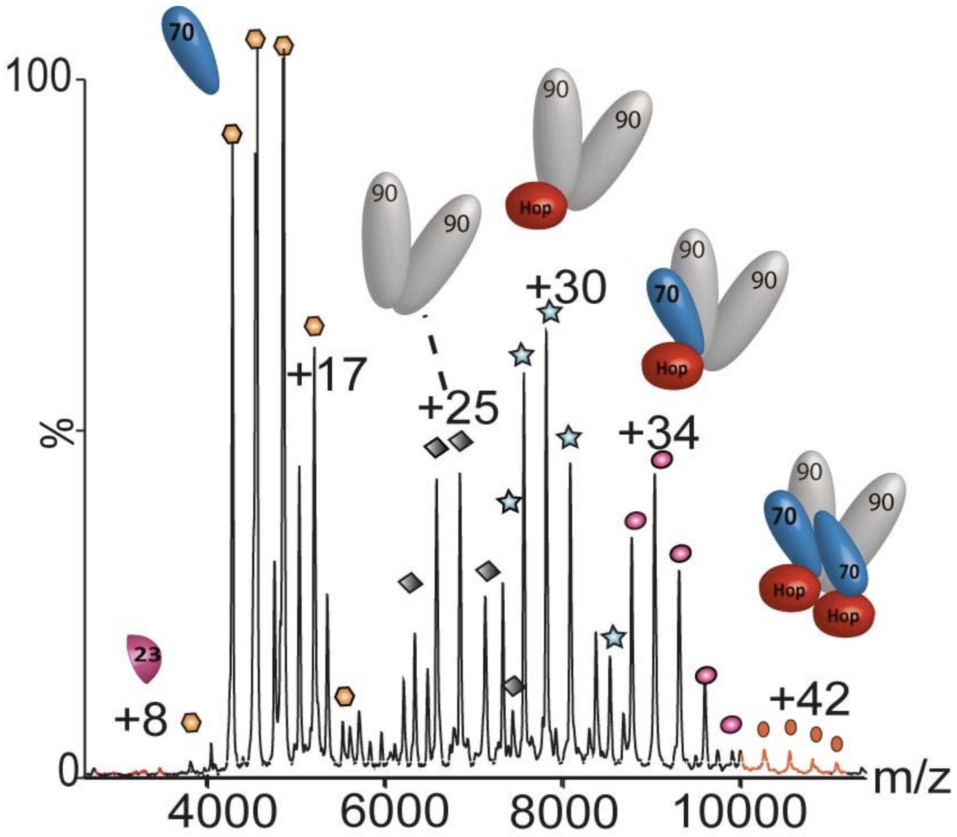

Figure S3: Examining p23 binding onto the Hsp90/Hop/Hsp70 complexes in the absence of the client protein GR. Data obtained for a solution containing equimolar $Hsp90_2$, Hop and $Hsp70_2$ (all at 1 µM) with 1 µM p23 in the presence of 300 µM ATP/Mg^{2+} and catalytic amounts of Hsp40. Spectrum shows no stable binding of p23 onto Hsp90 complexes in the absence of the client protein.

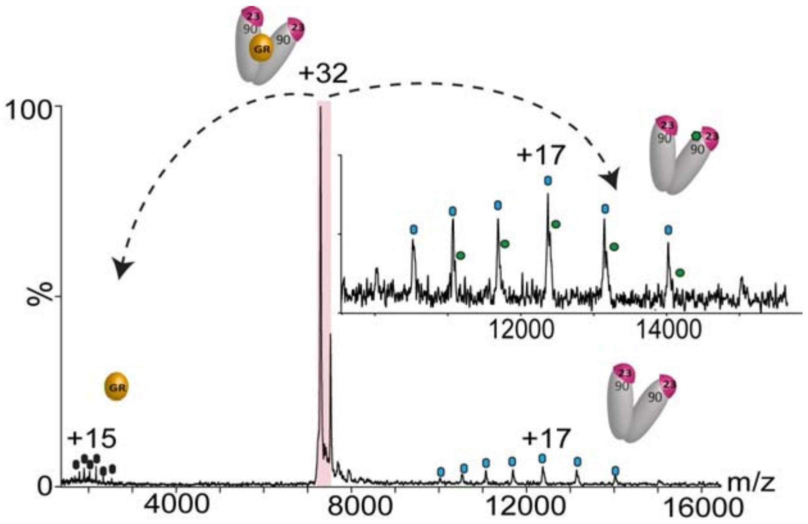

Figure S4: MS/MS dissociation of $(Hsp90)_2(GR)(p23)_2$. MS/MS dissociation of a 32+ charge state corresponding to the $(Hsp90)_2(GR)_1(p23)_2$ complex. Results show loss of GR (black circles) subunit releasing a stripped complex consisting of $(Hsp90)_2(p23)_2$ (light blue circles). Expansion of the stripped complex reveals a second charge state series corresponding to a single nucleotide bound to the stripped $(Hsp90)_2(p23)_2$ complex.

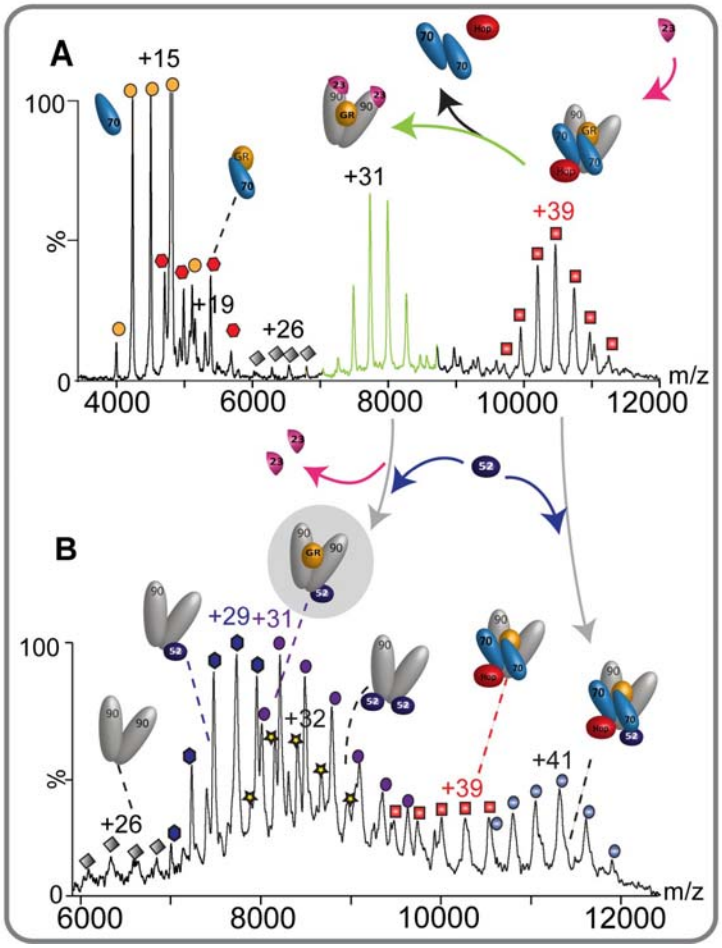

Figure S5: Addition of FKBP52 to p23-bound client complex. The formation of $(Hsp90)_2(GR)_1(p23)_2$ was investigated previously {Morgner, 2015 #134} and provides the starting point for investigating the effects of FKBP52. FKBP52 was added to a solution containing Hsp90/Hop/Hsp70/GR/Hsp40/p23. The complexes formed are the same as those formed when p23 and FKBP52 addition are reversed, with the exception of $(Hsp90)_2(GR)_1(FKBP52)_1(p23)_1$ which is not observed here.

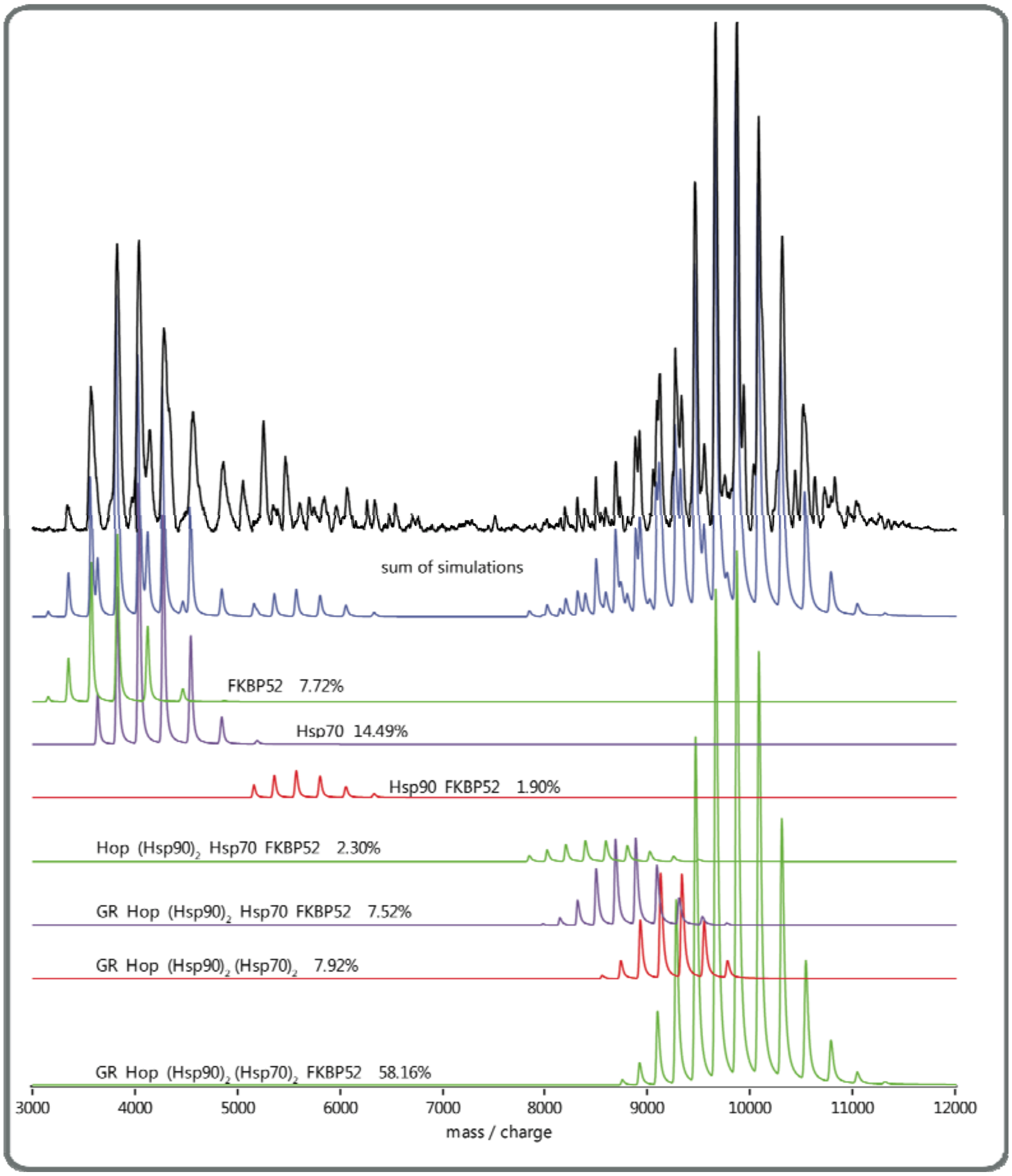

Figure S6: Assignment of the mass spectrum **Fig. 4A** and relative intensities of the complexes formed deduced by the program *Mass*ign [1].

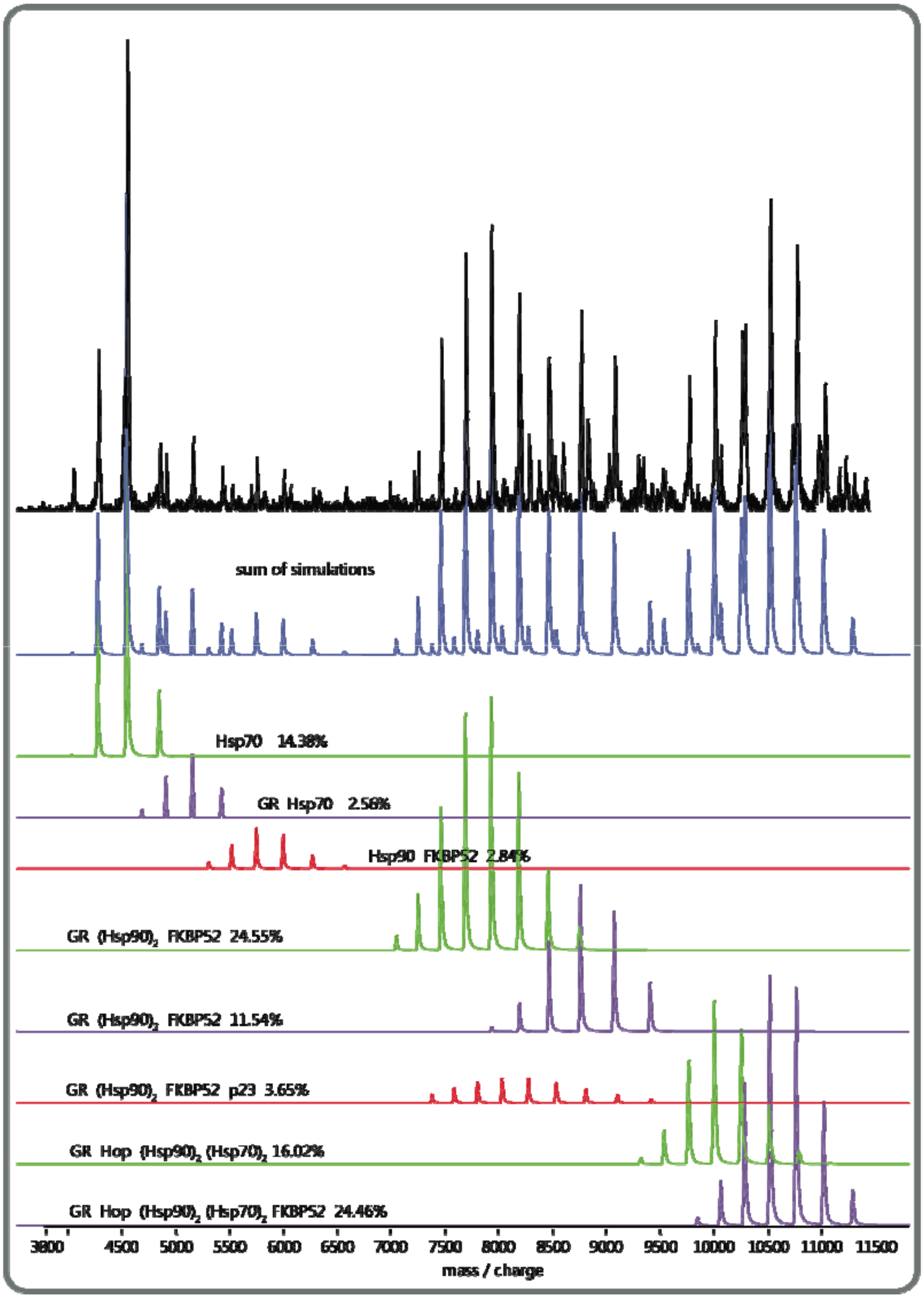

Figure S7: Assignment of the mass spectrum **Fig. 4B** and relative intensities of the complexes formed deduced by the program *Mass*ign [1].

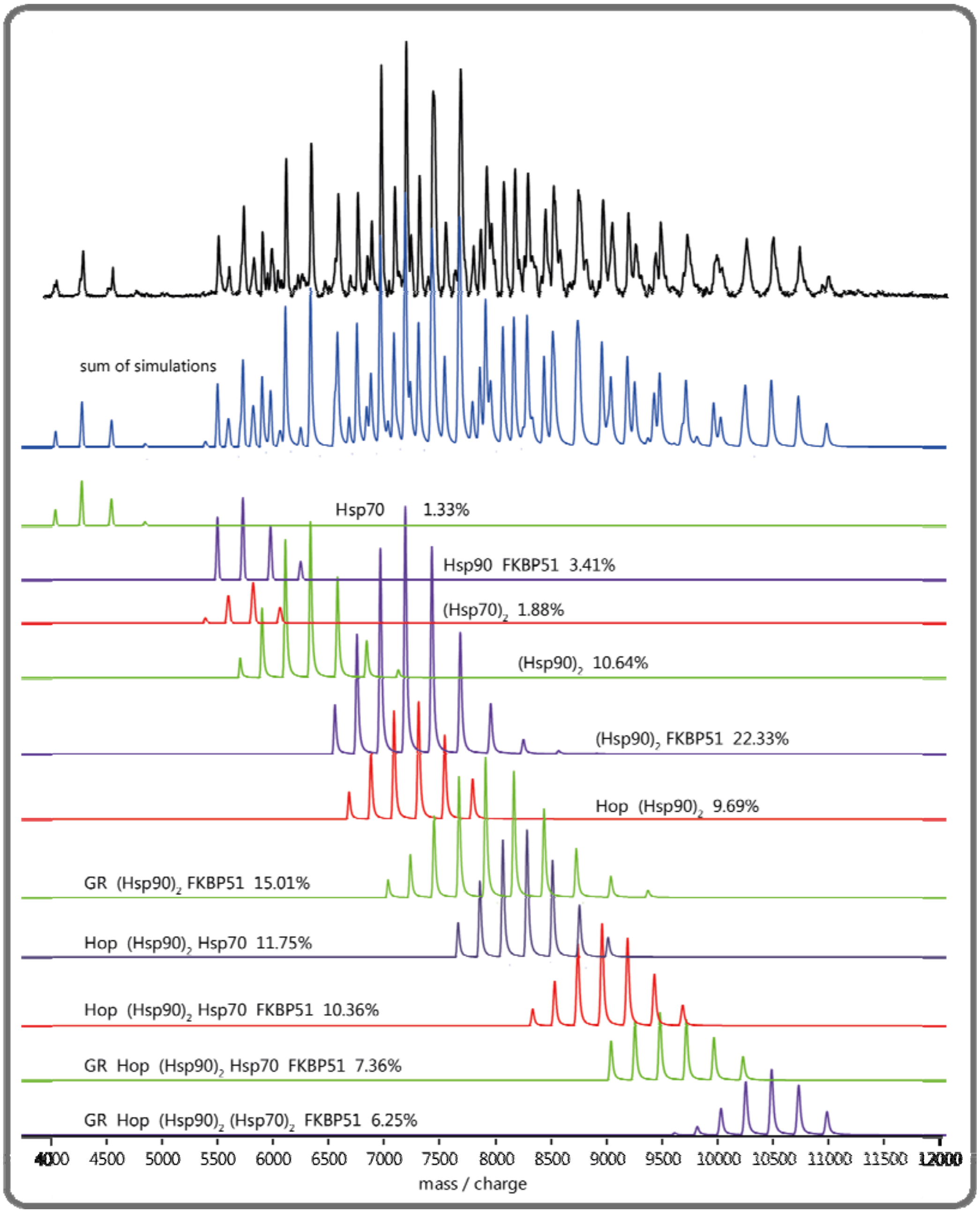

Figure S8: Assignment of the mass spectrum **Fig. 5A** and relative intensities of the complexes formed deduced by the program *Mass*ign [1].

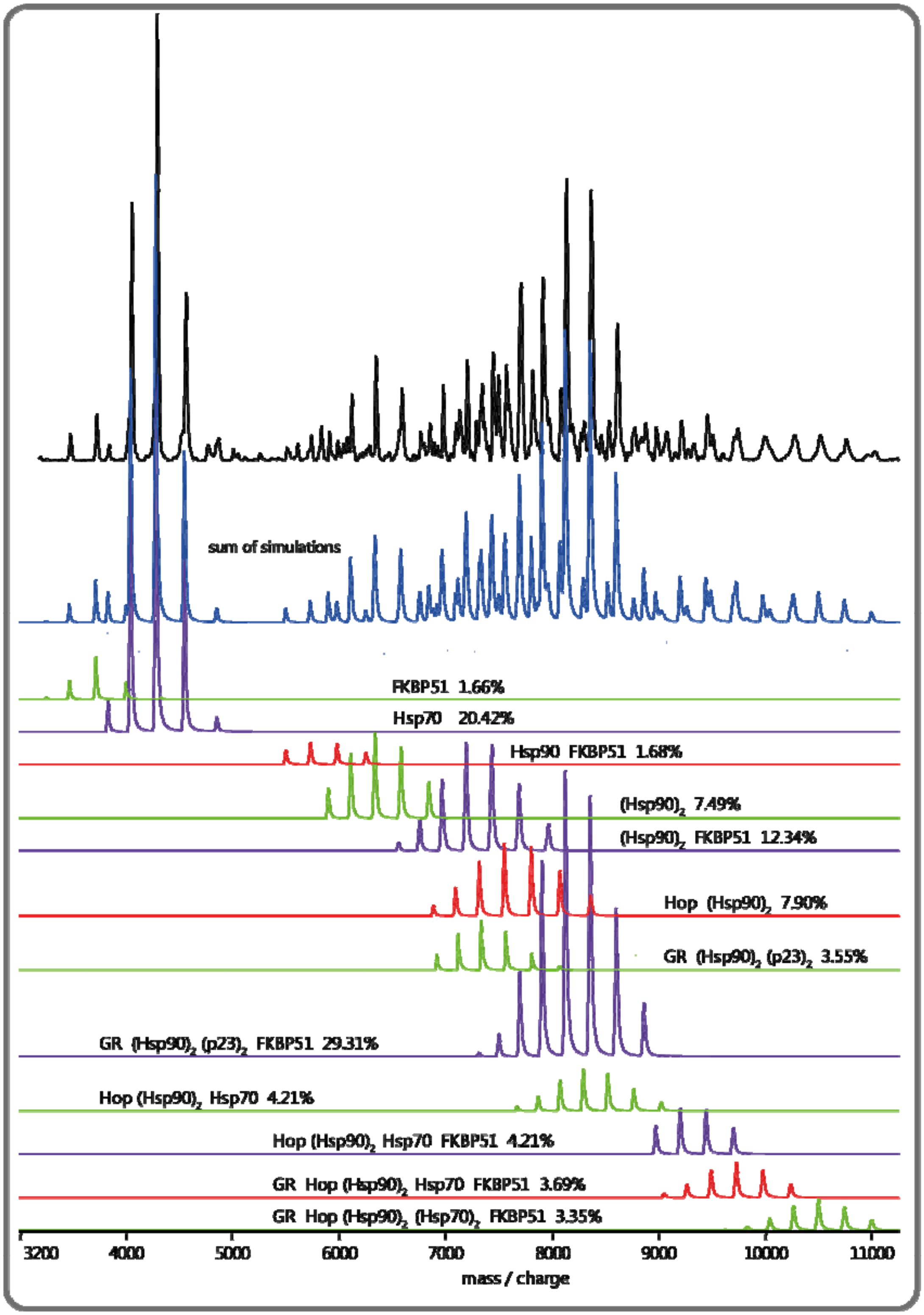

Figure S9: Assignment of the mass spectrum **Fig. 5B** and relative intensities of the complexes formed deduced by the program *Mass*ign [1].

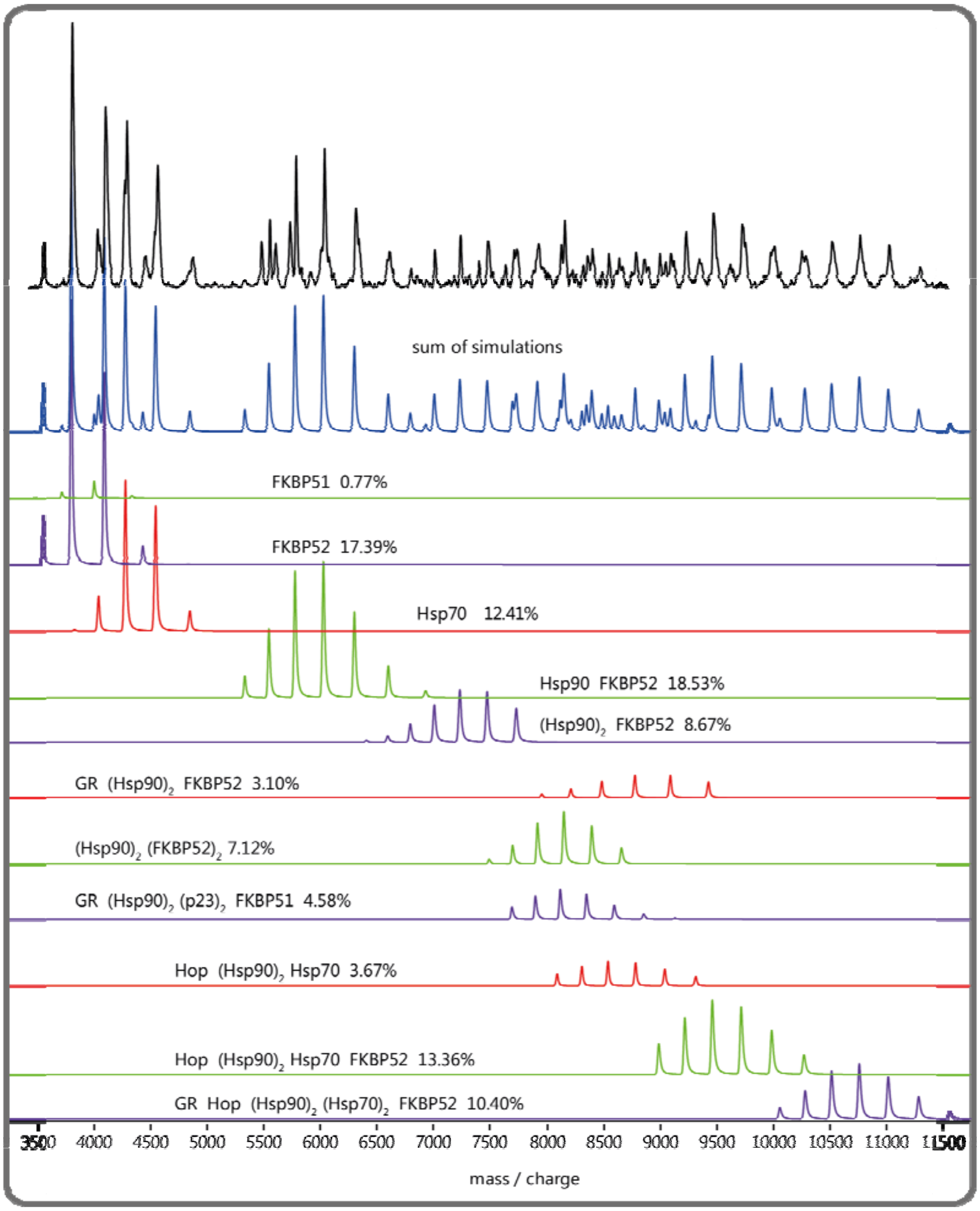

Figure S10: Assignment of the mass spectrum **Fig. 5C** and relative intensities of the complexes formed deduced by the program *Mas*sign [1].

Protein	Expected mass (Da)	Measured mass (Da)
p23	19468	19695 ± 50
GRLBD	30281	30291 ± 15
FKBP51	51810	52063 ± 21
FKBP52	52185	52705 ± 86
Hop	62738	63197 ± 49
Hsp70	72231	72673 ± 66
$(Hsp90)_1$	85443	85594 ± 32
$(Hsp70)_2$	144462	145393 ± 176
$(Hsp90)_2$	170887	171015 ± 26

Complexes	Expected mass (Da)	Measured mass (Da)
$(Hsp70)_1(GRLBD)_1$	102512	103024 ± 147
$(Hsp90)_1(FKBP51)_1$	137253	137493 ± 49
$(Hsp90)_1(FKBP52)_1$	137845	138166 ± 102
$(Hsp90)_1(FKBP51)_2$	189063	189196 ± 154
$(Hsp90)_1(FKBP51)_1(FKBP52)_1$	189655	189748 ± 16
$(Hsp90)_1(FKBP52)_2$	190247	190413 ± 41
$(Hsp90)_2(p23)_2$ [MSMS]	209822	211132 ± 56
$(Hsp90)_2(FKBP51)_1$	222696	222883 ± 75
$(Hsp90)_2(FKBP52)_1$	223228	223389 ± 195
$(Hsp90)_2(Hop)_1$	233625	233919 ± 79
$(Hsp90)_2(p23)_2(GRLBD)_1$	240103	240840 ± 60
$(Hsp90)_2(FKBP52)_1(GRLBD)_1$	253569	253682 ± 147
$(Hsp90)_2(FKBP52)_1(GRLBD)_1(p23)_1$	273037	273026 ± 266
$(Hsp90)_2(FKBP51)_2$	274506	274448 ± 186
$(Hsp90)_2(FKBP51)_1(FKBP52)_1$	275098	274603 ± 52
$(Hsp90)_2(FKBP52)_2$	275390	275292 ± 224
$(Hsp90)_2(Hop)_1(FKBP51)_1$	285434	285975 ± 54
$(Hsp90)_2(Hop)_1(FKBP52)_1$	286026	286676 ± 152
$(Hsp90)_2(p23)_2(GRLBD)_1(FKBP51)_1$	291913	292224 ± 146
$(Hsp90)_2(Hop)_2$	296363	296975 ± 181
$(Hsp90)_2(Hop)_1(Hsp70)_1$	305856	306489 ± 86
$(Hsp90)_2(Hop)_1(Hsp70)_1(FKBP51)_1$	357665	358384 ± 48
$(Hsp90)_2(Hop)_1(Hsp70)_1(FKBP52)_1$	358257	359408 ± 306
$(Hsp90)_2(Hop)_1(GRLBD)_1(Hsp70)_1(FKBP51)_1$	387946	388722 ± 40
$(Hsp90)_2(Hop)_1(GRLBD)_1(Hsp70)_1(FKBP52)_1$	388538	390799 ± 145
$(Hsp90)_2(Hop)_1(GRLBD)_1(Hsp70)_2$	408367	410078 ± 590
$(Hsp90)_2(Hop)_2(Hsp70)_2$	440825	442290 ± 200
$(Hsp90)_2(Hop)_1(GRLBD)_1(Hsp70)_2(FKBP51)_1$	460177	461442 ± 203
$(Hsp90)_2(Hop)_1(GRLBD)_1(Hsp70)_2(FKBP52)_1$	460769	462753 ± 51

Table S1: Calculated and experimentally measured masses of proteins and complexes formed.

	**Hsp70 (µM)**	**Hsp40 (µM)**	**GRLBD (µM)**	**FKBP51 (µM)**	**Hop (µM)**	**FKBP52 (µM)**	**P23 (µM)**	**Hsp90$_2$ (µM)**
Fig S1				**6**		**6**		**3**
Fig S2	0.5 &1	0.5 &1	0.5 &1	0.5 &1	0.5 &1	0.5 &1	-	0.5 &1
Fig S3	1	-	-	-	1	-	-	1
Fig S4	-	-	1	-	1	-	1	1
Fig S5	1	1	1	-	1	1	1	1

Table S2: protein concentrations for all experiments reported in the supplementary material.

1 **Morgner, N. & Robinson, C. V. Massign: an assignment strategy for maximizing information from the mass spectra of heterogeneous protein assemblies. *Anal Chem* 84, 2939-2948, doi:10.1021/ac300056a (2012).**

2 **Ebong, I. O. *et al.* Heterogeneity and dynamics in the assembly of the heat shock protein 90 chaperone complexes. *Proc Natl Acad Sci U S A* 108, 17939-17944, doi:10.1073/pnas.1106261108 (2011).**
